# Thermoresponsive Gel‐Yarn Composites Enable Low‐Temperature Tensile Actuation in Twisted and Coiled Polymer Artificial Muscles

**DOI:** 10.1002/marc.70432

**Published:** 2026-09-15

**Authors:** Sinmisola Aloko, Geoffrey Spinks, Javad Foroughi

**Affiliations:** ^1^ School of Mechanical and Manufacturing Engineering University of New South Wales Sydney Australia; ^2^ School of Biomedical Engineering University of Wollongong Wollongong New South Wales Australia; ^3^ Department of Cardiothoracic Transplantation, and Vascular Surgery Hannover Medical School Hannover Germany

**Keywords:** artificial muscles, fibre‐based actuators, gel‐yarn composites, low‐temperature actuation, thermoresponsive hydrogels, twisted and coiled polymer actuators

## Abstract

Soft robotic systems require actuators that combine large deformation, mechanical robustness, and reliable operation under practical conditions. Twisted and coiled polymer (TCP) artificial muscles, typically fabricated from fibrous materials, offer high power density and large stroke; however, their reliance on elevated operating temperatures (>80°C) for high performance limits their use in temperature‐sensitive environments. Here, we report a thermoresponsive gel‐yarn composite actuator that enables actuation at reduced temperatures within TCP‐based systems. Twisted polyester yarns were functionalized with a biocompatible thermosensitive hydrogel, poly(MAA‐co‐OEGMA), forming a fiber‐gel composite architecture. Temperature‐induced swelling and deswelling of the hydrogel in cold and hot water, respectively, generated internal stresses within the yarn, which drive untwisting of the coiled structure and result in lengthwise actuation. The effect of ionic crosslinking was systematically investigated by varying FeCl_3_ concentration, with 1 M identified as the optimal condition balancing structural stiffness and actuation performance. The composite actuators exhibit isotonic free strains of up to 39% when cycled between room temperature and 50°C and work densities of up to 8.8 × 10^−^^3^ J g^−^^1^, with reversible and repeatable actuation behavior. Compared to conventional TCP systems, which typically operate at substantially higher temperatures, the present system achieves similar deformation but within a lower and more practical thermal range. These findings suggest that fiber‐gel composite architectures provide a viable route for tuning actuation behavior in TCP systems while maintaining compatibility with temperature‐sensitive applications.

## Graphical abstract

Thermoresponsive gel–yarn composites integrate poly(MAA‐co‐OEGMA) hydrogels with twisted and coiled polyester yarns to enable reversible tensile actuation between 25°C and 50°C. Hydrogel swelling and deswelling generate internal stresses that drive lengthwise actuation, achieving isotonic strains up to 39%. Ionic crosslinking tunes mechanical and actuation performance, providing a promising strategy for low‐temperature artificial muscles.

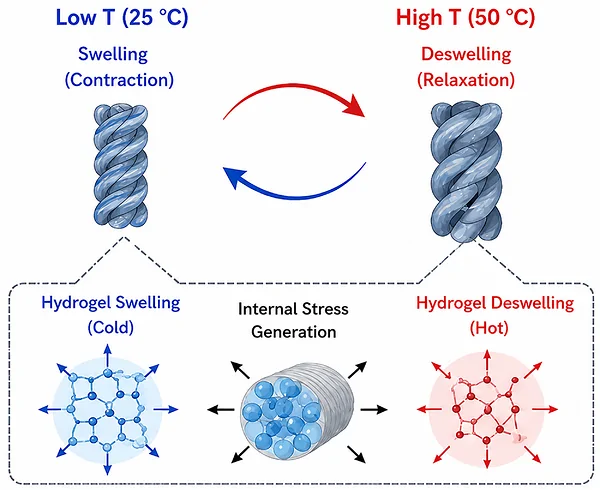

## 1 Introduction

The evolution of soft robotic systems has enabled safer and more compliant interaction with the human body compared to conventional rigid robotic systems. Their flexibility allows for safe interaction, mitigates safety concerns and compliance issues, and makes them suitable for a wide range of applications, including medical devices, manufacturing and automation, space exploration, and agriculture [1]. Artificial muscles have been widely used in soft robotic systems due to their ability to mimic the behavior of natural human muscles in response to various stimuli, such as heat, pressure, voltage, pH, or light. However, current artificial muscle technologies‐particularly twisted and coiled polymer (TCP) actuators‐are limited by high operating temperatures, relatively slow response, and reduced efficiency, necessitating continued development to overcome these challenges [2, 3].

The most reported materials for TCP actuators are thermoplastic fibers such as nylon (polyamide), polyester, polyethylene (PE), and polyvinylidene fluoride (PVDF), due to their ability to store torsional strain and exhibit thermally driven actuation. These TCP actuators can generate high power density and large stroke; however, they typically operate at elevated temperatures (> 80°C), which restricts their use in temperature‐sensitive environments, particularly in biomedical applications [4, 5]. To address this limitation, composite actuator systems have emerged as a promising technique, as they can combine the advantages of multiple material classes while mitigating their individual drawbacks [6, 7].

Thermosensitive hydrogels represent one such class of materials that can enable actuation at reduced temperatures. These materials undergo reversible volume phase transitions above their Lower Critical Solution Temperature (LCST), which can be tuned through polymer composition and molecular design. Examples include the widely studied poly(N‐isopropylacrylamide) (PNIPAM), with an LCST around 32°C, which makes it suitable for use in systems intended for biomedical environments. However, PNIPAM‐based systems often suffer from limited mechanical strength, motivating the development of modified or alternative thermoresponsive polymers [8, 9, 10, 11, 12, 13]. Recent studies have explored these materials as alternative actuators due to their ability to generate deformation through thermally induced structural changes [14, 15]. However, these systems often face challenges such as low mechanical strength and limited force output, restricting their standalone application [16, 17].

Integrating thermoresponsive hydrogels with fibrous architectures has therefore emerged as a promising way to couple low‐temperature responsiveness with structural load‐bearing capability [18]. Another important thermosensitive polymer is poly(methacrylic acid‐co‐oligo(ethylene glycol) methacrylate) (poly(MAA‐co‐OEGMA)), a comb‐graft polymer composed of methacrylic acid (MAA) and oligo(ethylene glycol) methacrylate (OEGMA). Compared to PNIPAM, poly(MAA‐co‐OEGMA) offers improved biocompatibility, enhanced hydrolytic stability, and reduced protein adsorption, making it particularly suitable for biomedical and soft actuation applications [19]. MAA is an acidic monomer that is hydrophobic at low pH, while OEGMA is hydrophilic and thermosensitive; their combination provides tunability in the resulting polymer by adjusting the polymer composition [20].

In fiber‐gel composite systems, the incorporation of thermosensitive hydrogels into twisted and coiled yarn structures can generate internal stresses during swelling and deswelling, driving untwisting of the coiled configuration and producing mechanical actuation. However, achieving efficient coupling between hydrogel swelling behavior and fiber mechanics, while maintaining structural integrity and actuation performance, remains a significant challenge.

In this study, we report a thermoresponsive gel‐yarn composite actuator based on a poly(MAA‐co‐OEGMA) hydrogel integrated within twisted polyester yarns (Figure 1). By systematically tuning ionic crosslinking through FeCl_3_ concentration, we investigate the relationship between composite structure, mechanical properties, and actuation performance. This approach enables high‐stroke, reversible actuation at reduced temperatures while retaining significant deformation, providing a scalable materials strategy for low‐temperature TCP‐based artificial muscles.

**FIGURE 1:**
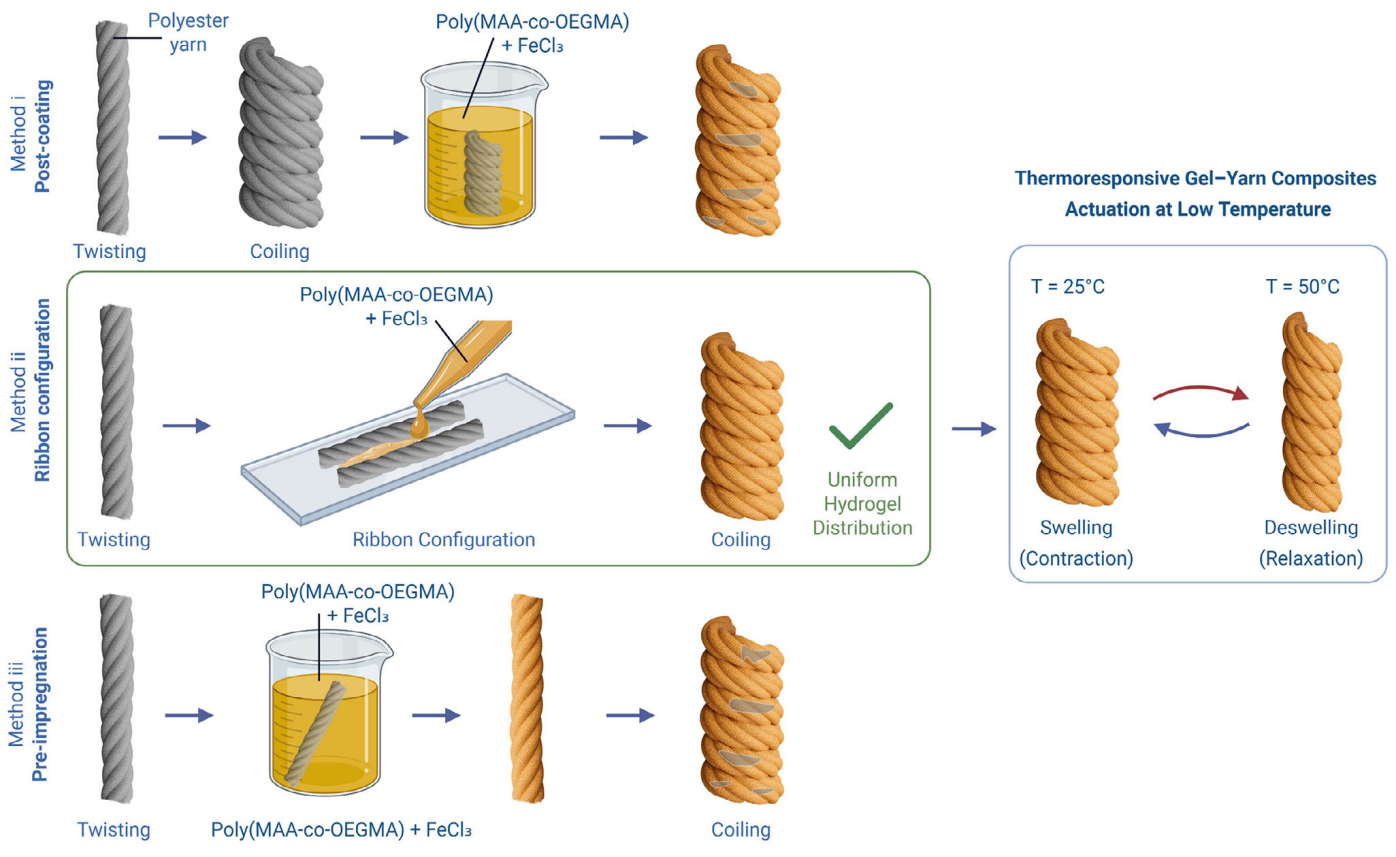
Fabrication strategies and actuation mechanism of thermoresponsive hydrogel‐TCP composite actuators. Three hydrogel incorporation methods are illustrated: (i) post‐coating after twisting and coiling, (ii) ribbon configuration, and (iii) pre‐impregnation. In the ribbon configuration (highlighted), hydrogel is introduced between parallel yarns prior to twisting, resulting in improved retention and uniform hydrogel distribution within the coiled structure.

## 2 Materials and Methods

### 2.1 Hydrogel Formulation

The hydrogel composition was based on the formulation reported by Jiang et al. [21], consisting of 76% methacrylic acid (MAA) and 24% oligo(ethylene glycol) methacrylate (OEGMA_500_). Increasing the MAA content has been shown to enhance the mechanical strength of the resulting hydrogel; however, concentrations exceeding 80% may lead to the formation of insoluble complexes. A polymer concentration of 300 mg mL^−^^1^ was selected, as it exhibits a reversible sol‐gel transition between 25°C and 50°C. This concentration allows for sufficient network formation while maintaining processability. The resulting hydrogels exhibit anisotropic and aligned structures, which are advantageous for directional actuation.

The lower critical solution temperature (LCST) of poly(MAA‐co‐OEGMA) is influenced by both polymer composition and molecular weight. Poly(MAA_140_‐co‐OEGMA_99_), with an approximate molecular weight of 51 000 g mol^−^^1^, was employed due to the presence of carboxylic functional groups that facilitate ionic crosslinking and enable further functionalization. Hydrogen bonding between MAA and OEGMA contributes to network cohesion, while minor imine bond formation (approximately 0.01–2 mol%) provides additional stabilization of the hydrogel structure. Iron(III) chloride (FeCl_3_) was selected as the primary crosslinking agent. Alternative crosslinkers such as tannic acid and calcium chloride have been reported; however, FeCl_3_ was chosen due to its ability to provide effective ionic crosslinking and improved structural integrity of the gel network [21].

### 2.2 Polymerization

Poly(MAA‐co‐OEGMA) was synthesized via a one‐pot reversible addition‐fragmentation chain transfer (RAFT) polymerization based on the method reported by Jiang et al. [21], where a precursor solution was prepared by dissolving 12 mL MAA, 18 mL OEGMA_500_, and 56 mg azobisisobutyronitrile (AIBN) as the radical initiator in 70 mL anhydrous dimethylformamide (DMF) in a sealed glass flask. The solution was purged with nitrogen gas for 10 min to remove dissolved oxygen and prevent premature termination of radical polymerization. The reaction mixture was then maintained at 65°C for 24 h. Following polymerization, the solution was cooled in an ice bath (0°C) and exposed to air. The viscous polymer solution was precipitated three times in diethyl ether to remove residual monomers and solvent impurities. The purified polymer was collected and stored for further use. The resulting bulk polymer solution had a concentration of approximately 300 mg mL^−^^1^.

### 2.3 Hydrogel Preparation

For hydrogel preparation, 0.5 mL of the 300 mg mL^−^^1^ polymer stock solution was mixed with 1.0 mL deionized water to reduce viscosity and improve coating uniformity. The diluted polymer solution was mixed until visually homogeneous before the addition of the crosslinker. A 10 µL aliquot of an aqueous FeCl_3_ stock solution was then added to induce ionic crosslinking. FeCl_3_ stock concentrations of 0.1, 0.25, 1, and 2 M were investigated, while the uncrosslinked control contained no FeCl_3_. Unless otherwise stated, the FeCl_3_ concentrations used throughout this manuscript refer to the concentration of the FeCl_3_ stock solution from which a 10 µL aliquot was added, rather than the final FeCl_3_ concentration in the hydrogel formulation. The resulting formulations were denoted according to the FeCl_3_ stock concentration used, namely Gel 0, Gel 0.1, Gel 0.25, Gel 1, and Gel 2. The formulations were thoroughly mixed immediately before application to the polyester yarns. No separate curing step was performed before coating; ionic crosslinking and structural stabilization continued during composite preparation and subsequent drying at 70°C for 10 min. The resulting hydrogel formulations were denoted as Gel X.

### 2.4 Swelling Characterization

Hydrogel samples were cast in Teflon molds (10 mm × 10 mm) and dried at 70°C. The dried samples were weighed and immersed in deionized water to reach equilibrium swelling. Maximum swelling was achieved after 24 h at room temperature. Swelling‐deswelling behavior was evaluated by alternately exposing samples to 25°C and 50°C at 5 min intervals. Prior to weighing, excess surface water was removed to ensure measurement consistency.

### 2.5 Gel‐Yarn Composite Fabrication

Three fabrication strategies were evaluated to determine the optimal hydrogel incorporation method:

- coating after twisting and coiling,
- ribbon configuration (hydrogel deposited between parallel yarns prior to twisting), and
- coating after twisting followed by coiling.

All samples were dried in the oven at 70°C for 10 min.

The gel‐to‐yarn weight ratio was calculated using Equation (1):

(1) $$\mathrm{Gel}/\mathrm{Yarn}\;\mathrm{ratio}\;=\frac{W_{\mathrm{composite}}-W_{\mathrm{yarn}}}{W_{\mathrm{yarn}}}$$

The ribbon configuration (method ii) was selected for subsequent experiments due to improved gel retention and uniform distribution. Two polyester yarns (length: 150 mm) were aligned in parallel with a spacing of approximately 2 mm on a Teflon substrate. A volume of 150 µL of Gel X was deposited to form a coating reservoir, and the yarns were passed through the gel solution to ensure uniform coating along their length. The coated yarns were subsequently twisted and coiled, and a small weight was attached to maintain tension and prevent entanglement during processing. The samples were dried at 70°C for 10 min to evaporate water and fix the coiled structure. This temperature was selected based on preliminary studies, where lower temperatures were insufficient for coil fixation and temperatures above 90°C led to degradation [22].

The gel content in the composite was calculated using Equation (2):

(2) $$\mathrm{Gel}\;\left(\%\right)=\frac{W_{\mathrm{dry}\;\mathrm{composite}}-W_{\mathrm{dry}\;\mathrm{yarn}}}{W_{\mathrm{dry}\;\mathrm{yarn}}}\;\times 100$$

A volume of 150 µL was identified as optimal, as higher volumes resulted in excess surface gel, which interfered with actuation and caused adhesion between adjacent coils.

### 2.6 Actuation Analysis

Actuation behavior was evaluated under a temperature change of ΔT = 25°C (25°C–50°C), corresponding to the swelling‐deswelling transition of the hydrogel across its LCST. The swollen state at 25°C was defined as the actuated state, while the deswollen state at 50°C was defined as the unactuated state. Testing was performed by conditioning the sample at each temperature to ensure equilibrium swelling was attained. Mechanical testing was performed using an Instron 5566 tensile testing system (Illinois Tool Works) with Bluehill software for data acquisition. Samples were mounted vertically using mechanical grips and immersed in temperature‐controlled water baths.

Samples were subjected to a pre‐strain of 7.5%, and testing was conducted under cyclic conditions. Each test cycle consisted of:

- preconditioning in hot water (50°C) for 20 min,
- isometric actuation in water at 25°C for 40 min, and
- recovery in hot water (50°C) to assess reversibility and creep behavior.

Load‐unload curves were obtained under both actuated (25°C) and unactuated states (50°C), and force‐displacement data were recorded for subsequent analysis.

### 2.7 Scanning Electron Microscopy (SEM)

Morphological characterization of the gel‐yarn composites was performed using a JEOL JCM‐6000 SEM. Samples were oven‐dried prior to imaging to remove residual moisture. Control samples consisting of untreated polyester yarn were also prepared for comparison. Samples were mounted on carbon tape to ensure conductivity, and images were acquired at magnifications of ×50, ×85, and ×200.

### 2.8 Differential Scanning Calorimetry

Thermal analysis was performed using a DSC 214 Polyma (Netzsch, Germany) to determine the LCST behavior of the composites. Samples were equilibrated in water for 24 h prior to testing to ensure full swelling. Sample masses ranged from 6.5 to 7.5 mg. Thermal measurements were conducted over a temperature range of 0°C–60°C at a heating rate of 0.5 K min^−^^1^. The resulting thermograms were analyzed to identify phase transition behavior.

### 2.9 Statistical Analysis

All quantitative data are presented as mean ± standard deviation. Statistical comparisons between groups were performed using one‐way analysis of variance (ANOVA), with differences considered statistically significant at p < 0.05. Data analysis and curve fitting were carried out using standard numerical methods to ensure consistency and reproducibility of the extracted actuation parameters.

## 3 Results

### 3.1 Thermoresponsive Swelling and Reversible Volume Transition of poly(MAA‐co‐OEGMA) Hydrogel

The poly(MAA‐co‐OEGMA) hydrogel exhibited pronounced thermoresponsive swelling behavior under room‐temperature conditions (25°C), with the swelling ratio compared to the dry state increasing progressively from 170% at 5 min to 669% at 25 min (Table 1). Upon transfer to heated water (50°C), the hydrogel underwent rapid deswelling, showing a reproducible reduction in swollen mass ranging from 17% to 35% across the tested time points. Representative images further show the reversible nature of this response, with the gel expanding substantially during incubation in room‐temperature water and contracting following thermal stimulation before reswelling upon re‐exposure to 25°C conditions. (Figure 2). The repeatable swelling‐deswelling transition across sequential thermal cycles confirms the hydrogel's reversible volumetric response and supports its suitability as a candidate material for subsequent soft actuation studies.

**TABLE 1:** Time‐dependent swelling and thermally induced deswelling behavior of poly(MAA‐co‐OEGMA) hydrogel.

Time (mins)	Swelling (%)	Deswelling (%)
5	170	32
10	223	29
15	348	31
20	485	35
25	669	17

**FIGURE 2:**
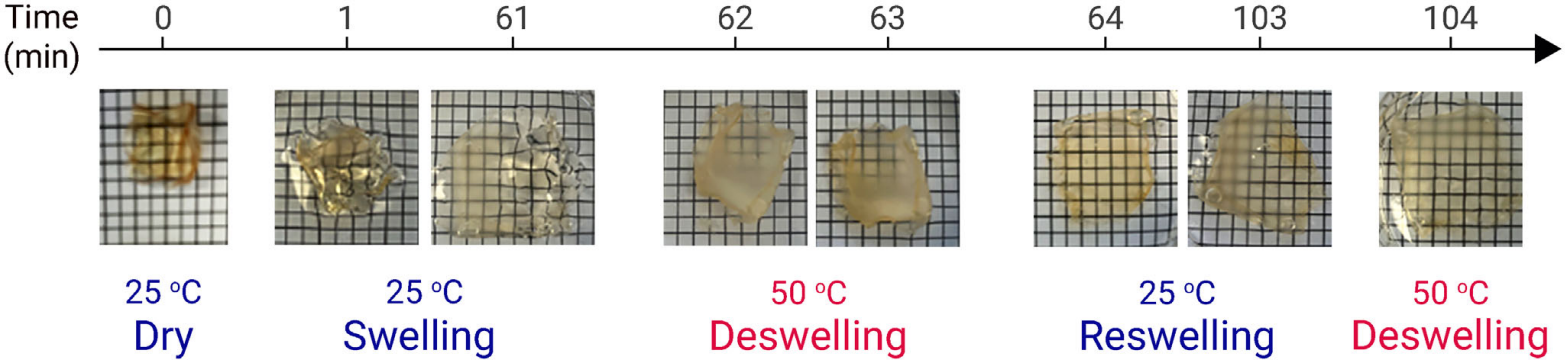
Representative sequential images showing reversible swelling‐deswelling behavior of the hydrogel under alternating 25°C and 50°C conditions.

### 3.2 Mechanical Behavior of FeCl_3_‐Crosslinked Gel‐Yarn Composites

To investigate the effect of ionic crosslinking density on the mechanical response of the gel‐yarn composites, representative cyclic load‐extension curves were compared across samples prepared using FeCl_3_ stock concentrations ranging from 0 to 2 M (Figure 3). The tensile behavior of the composites was measured at both 25°C and 50°C and plotted on the same axes with the origin corresponding to the unloaded / unactuated (50°C) condition. The horizontal and vertical distances between the two load‐extension curves can then indicate the actuation stroke and force generated, respectively, as detailed previously [23]. The load‐extension profiles demonstrated a clear dependence on crosslinker concentration, with more highly crosslinked samples exhibiting smaller separation between the 50°C and 25°C curves and steeper loading curves, indicative of increased network stiffness. In particular, the 2 M FeCl_3_ sample showed closely spaced stretch curves at the two temperatures and limited extension, suggesting that excessive crosslinking constrained reversible actuation.

**FIGURE 3:**
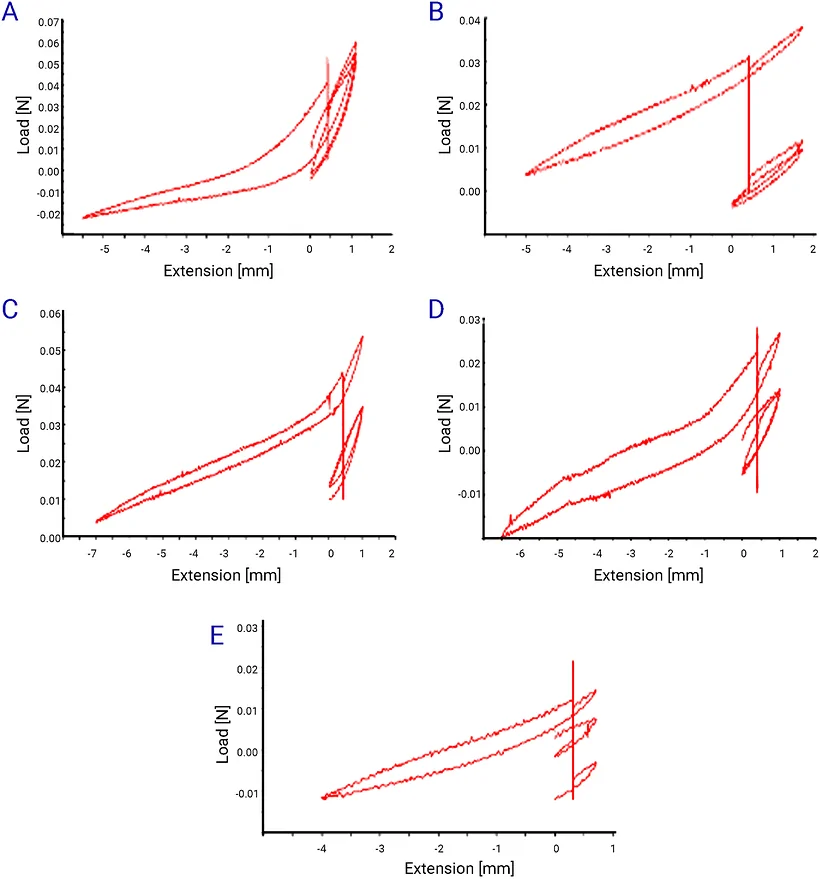
Mechanical response of gel‐yarn composites as a function of FeCl_3_ crosslinker concentration. Representative cyclic load‐extension curves of gel‐yarn composite samples prepared using different FeCl_3_ stock concentrations: (A) 2 M, (B) 1 M, (C) 0.25 M, (D) 0.1 M, and (E) 0 M (polymer only). Increasing crosslinker concentration altered both the stiffness and reversible deformation behavior of the composites.

At intermediate FeCl_3_ concentrations (1 and 0.25 M), the hydrogel actuators displayed more balanced mechanical behavior, characterized by larger actuation strokes and reproducible loading‐unloading hysteresis over repeated cycles. These samples retained highly consistent initial and repeat stretch profiles, indicating enhanced structural stability and reliable cyclic deformation under mechanical testing. By contrast, the 0.1 M FeCl_3_ group exhibited greater variation between repeated cycles, reflecting reduced network integrity at lower crosslinking density. The polymer‐only sample without FeCl_3_ (0 M) showed the largest deviation between initial and repeated stretch cycles, confirming poor mechanical repeatability in the absence of ionic crosslinking.

Figure 3 demonstrates that FeCl_3_ concentration strongly influenced the cyclic load‐extension behavior of the gel‐yarn composites. The 2 M FeCl_3_ samples (Figure 3A) exhibited the steepest loading slopes and generated loads of up to approximately 60 mN, corresponding to stiffness values of 0.047–0.089 N mm^−^^1^. However, the available reversible deformation was comparatively restricted. By contrast, the stiffness values remained broadly similar across the 0–1 M formulations, generally within approximately 0.007–0.026 N mm^−^^1^. The 1 M composites (Figure 3B) sustained loads of approximately 40 mN while retaining a broader separation between the actuated and unactuated curves, consistent with greater reversible actuation. The 0.25 and 0.1 M samples (Figure 3C,D) show more compliant responses and greater sample‐to‐sample variability, whereas the uncrosslinked composites (Figure 3E) exhibited the least consistent recovery between repeated measurements. Loading‐unloading separation was observed across the formulations, indicating hysteretic mechanical behavior during cyclic deformation.

### 3.3 Gel‐Yarn Composites

Among the three fabrication strategies evaluated, the ribbon configuration produced the most uniform hydrogel incorporation and coil integrity after drying. The resulting guest‐host composite maintained a continuous coiled geometry, indicating efficient retention of the hydrogel phase within the twisted polyester host and enabling subsequent dimensional contraction through fiber untwisting (Figure 4).

**FIGURE 4:**
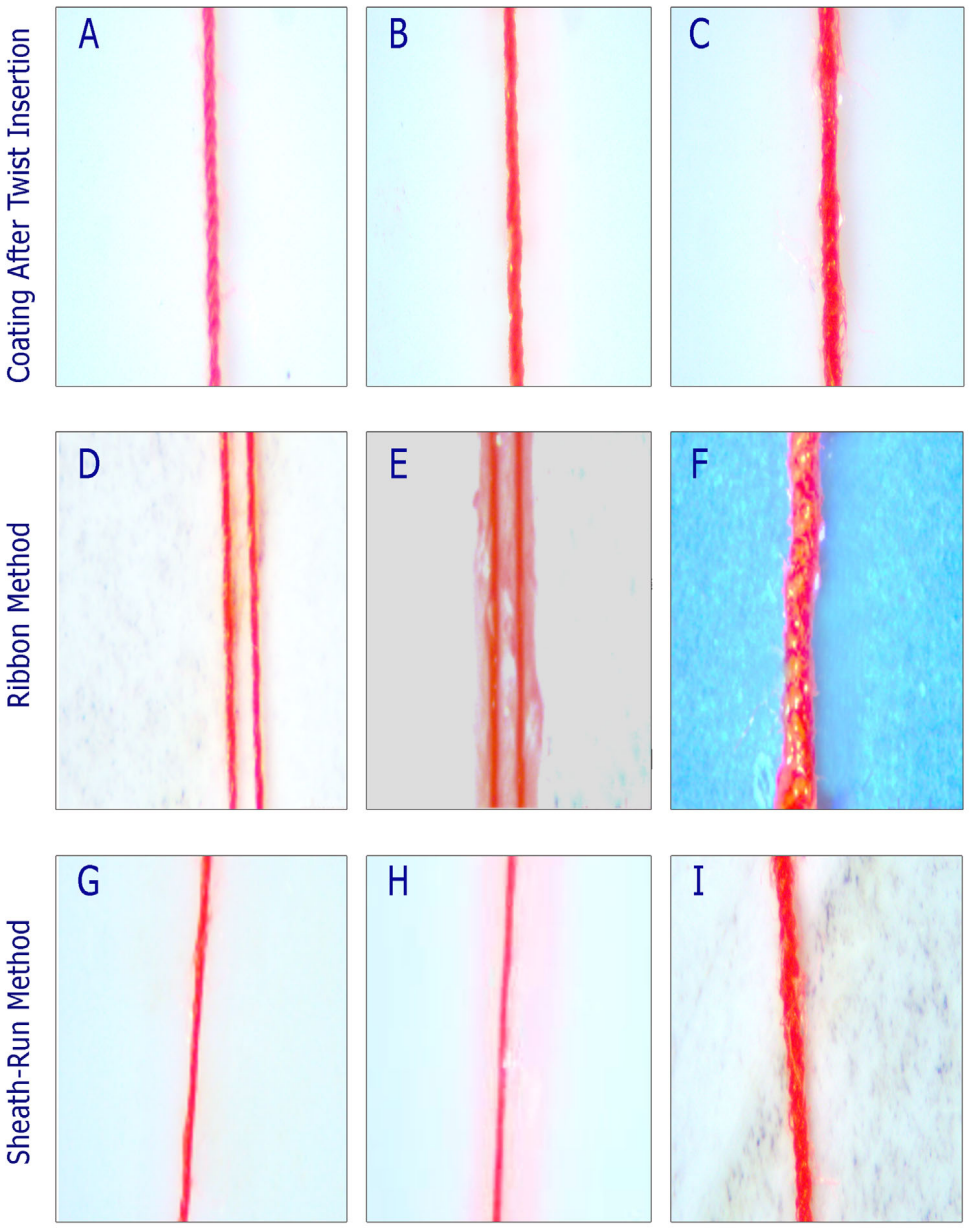
Optical microscopy images of gel‐yarn composites fabricated via (i) coating after twist insertion (A–C), (ii) the ribbon method (D–F), and (iii) the sheath‐run method (G–I).

Hydrogel incorporation into polyester yarns resulted in infiltration within the filament bundles, with minimal visible surface accumulation (Figure 5). Among the evaluated fabrication strategies, the ribbon configuration enabled uniform gel distribution and effective retention within the yarn structure. The twisted and coiled composites retained their structure following thermal setting at 70°C and remained stable during thermal cycling. Gel‐yarn composites were made from polymer solutions of 0, 0.1, 0.25, 1, and 2 M iron chloride as the crosslinker.

**FIGURE 5:**
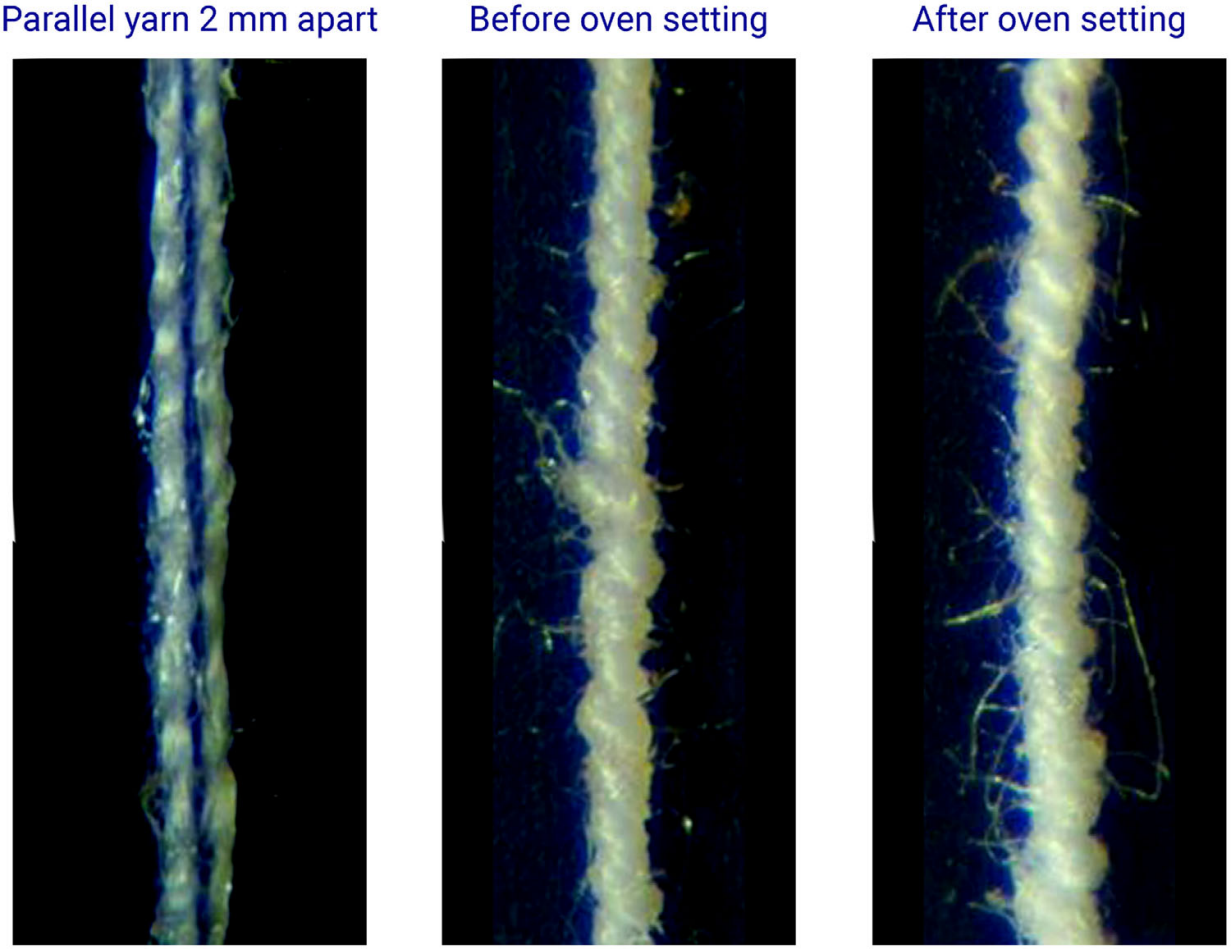
Representative fabrication sequence of composite sample X3. From left to right: parallel yarns following immersion in the polymer solution; the composite after twist insertion and coiling; and the final twisted and coiled composite after thermal setting at 70°C for 10 min.

### 3.4 Actuation Analysis

The actuation performance of the gel‐yarn composite actuators was evaluated using a force‐displacement analysis based on the methodology reported by Spinks et al. [23]. This method enables the extraction of key actuation parameters, including isotonic displacement and force generation, through comparison of the tensile mechanical response in the actuated and unactuated states.

Prior to testing, samples were immersed in room‐temperature water for 24 h to achieve equilibrium swelling. The swollen samples were then mounted on an Instron mechanical testing system, with one end fixed and the other subjected to controlled tensile loading. Load‐extension curves were recorded under two thermal conditions: elevated temperature (50°C), corresponding to the deswollen (unactuated) state, and room temperature (25°C), representing the swollen (actuated) state. To assess reversibility and repeatability, samples were cycled between thermal environments, including re‐equilibration in room‐temperature water for 40 min followed by reheating at 50°C for 20 min prior to a repeat stretch (7.5% strain).

The raw load‐extension data were processed by fitting polynomial trendlines to reduce experimental noise and obtain smooth representative curves (Figure 6A–D). As all curves showed considerable loading/unloading hysteresis (Figure 6A,C) the polynomial fits were used to create a single load‐extension curve that represented the average between the loading and unloading limits (Figure 6E). The average load‐extension curves for both the actuated and unactuated states were subsequently used to calculate the isometric actuation force generated at any pre‐load by taking the vertical distance between the two curves. This dataset was then transformed into extension‐load coordinates (Figure 6F), enabling direct determination of isotonic displacement as a function of applied preload as the horizontal distance between the two temperature curves. This analysis framework provides a quantitative description of thermally induced actuation and facilitates comparison between different composite configurations.

**FIGURE 6:**
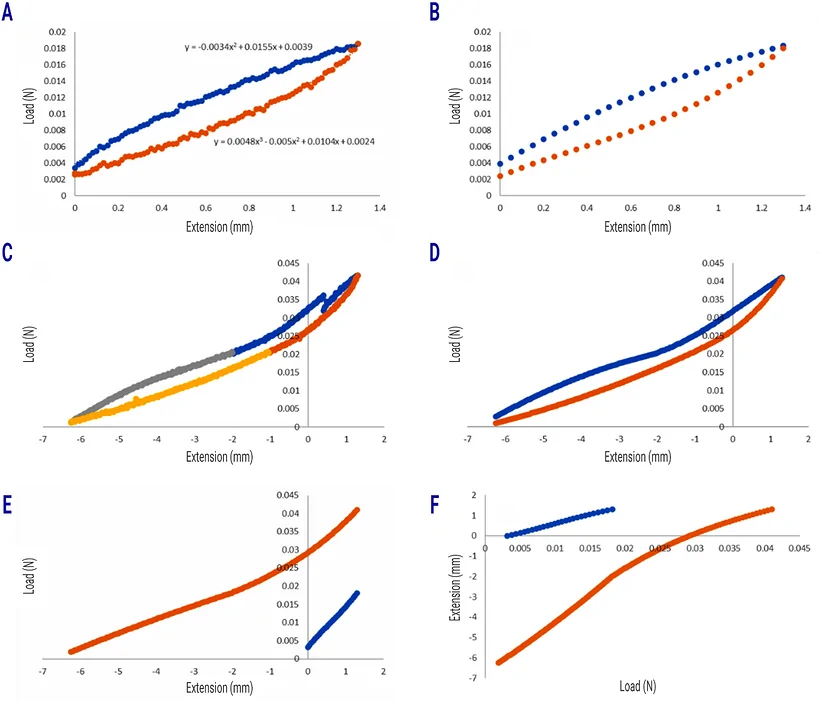
Quantitative extraction of actuation behavior from load‐extension analysis of gel‐yarn composite actuators prepared using 1 M FeCl_3_. (A) Representative raw load‐extension curves for the unactuated, deswollen state at 50°C, with blue indicating loading and orange indicating unloading. (B) Corresponding polynomial fits to the loading and unloading curves in panel A, using the same color coding. (C) Representative raw load‐extension curves for the actuated, swollen state at 25°C; the grey and blue segments represent the loading trace, whereas orange represents unloading. (D) Corresponding polynomial fits to the actuated loading and unloading curves, shown in blue and orange, respectively. (E) Averaged load‐extension curves obtained from the processed datasets, with blue representing the unactuated state at 50°C and orange representing the actuated state at 25°C. The origin was aligned with the unloaded, unactuated condition. (F) Transformation of the averaged curves to an extension‐load representation. The horizontal separation between the unactuated and actuated curves at a given preload represents the isotonic displacement.

### 3.5 Effect of Crosslinking Density on Actuation Performance

The load‐extension curves of gel‐yarn composite actuators prepared with varying FeCl_3_ concentrations are presented in Figure 7. For all samples, a measurable shift between the unactuated (50°C) and actuated (25°C) curves was observed, indicating thermally induced contraction by cooling of the composite structures. The magnitude of this shift varied across samples, reflecting differences in isotonic displacement at comparable preload forces.

**FIGURE 7:**
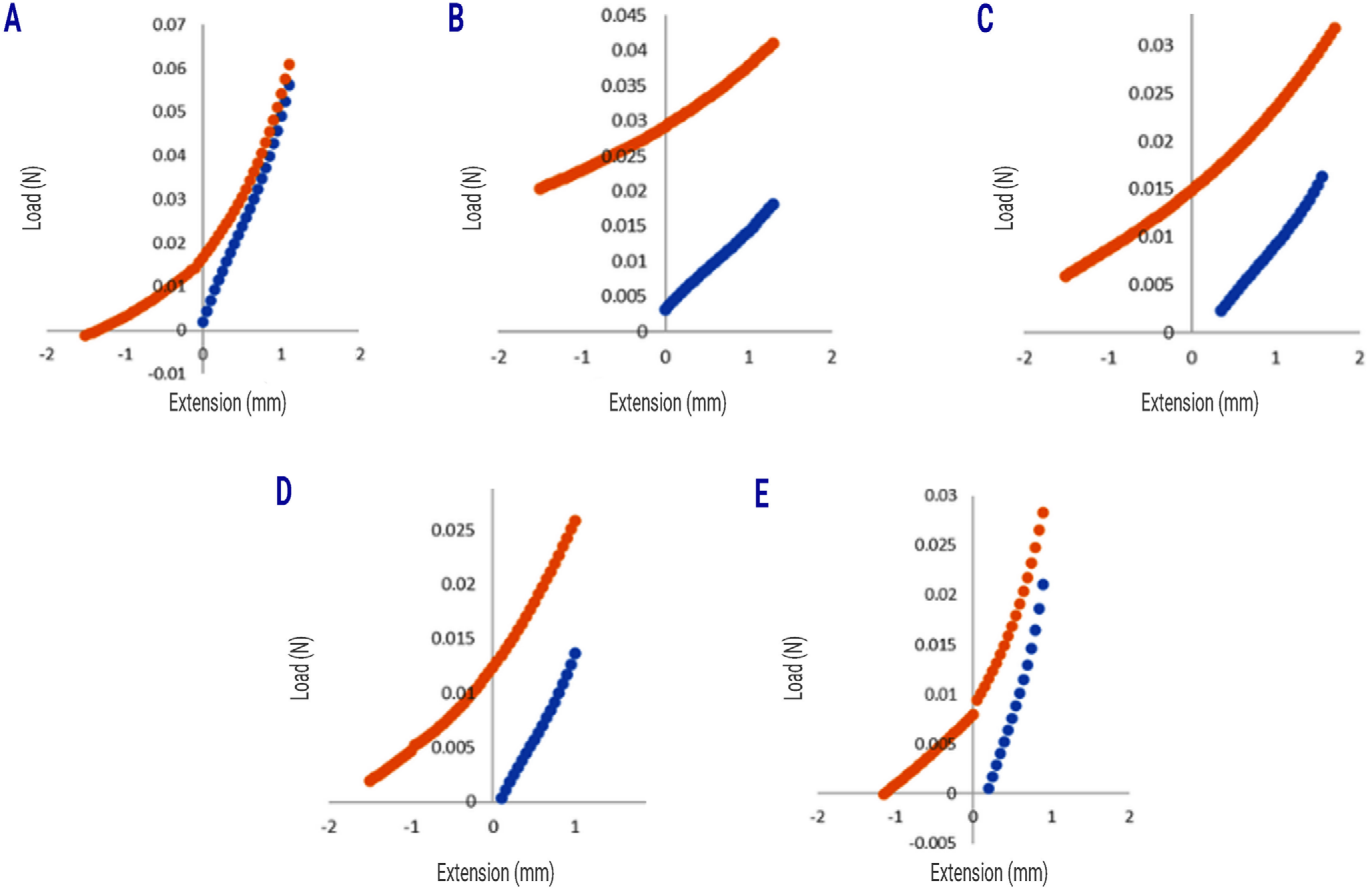
Load‐extension behavior of gel‐yarn composite actuators with varying FeCl_3_ crosslinker concentrations. Representative averaged extension‐load plots for composite samples prepared with different FeCl_3_ concentrations: (A) 2 M, (B) 1 M, (C) 0.25 M, (D) 0.1 M, and (E) 0 M. The curves correspond to the actuated (swollen, 25°C) and unactuated (deswollen, 50°C) states.

The 2 M FeCl_3_ sample (Figure 7A) exhibited the smallest separation between the actuated and unactuated curves. However, the 1 and 0.25 M samples (Figure 7B,C) showed a larger separation between curves over the tested extension range. The 0.1 M FeCl_3_ sample (Figure 7D) showed reduced separation with increased variability in curve profiles. The polymer‐only sample (0 M; Figure 7E) displayed the least consistent behavior, with minimal and irregular separation between the actuated and unactuated states. Variations in the load‐extension profiles and the separation between curves were observed across the different crosslinker concentrations, indicating differences in actuation response under identical testing conditions. The load–extension plots for all composite samples are presented in Figure 7.

### 3.6 Isotonic Strain and Work Output

The isotonic strain was calculated over a range of applied preload forces using the processed load‐extension data for each sample. The polynomial trendline equations obtained from the fitted curves were used to determine the corresponding actuated and unactuated lengths at each preload force, allowing calculation of isotonic displacement. The isotonic strain was then expressed as the percentage change in length relative to the original unactuated sample length.

The variation of isotonic strain with preload force is shown in Figure 8 for the sample crosslinked with 1 M FeCl_3_. The magnitude of isotonic strain varied with increasing preload force, with a consistent decrease in contraction observed as the pre‐load force increased. The work done per contraction cycle was calculated for each preload condition by multiplying the applied preload force by the corresponding isotonic displacement. The relationship between work output normalized to the sample mass and the preload force is also presented in Figure 8, showing variation in work done per cycle over the tested force range for the representative composite sample.

**FIGURE 8:**
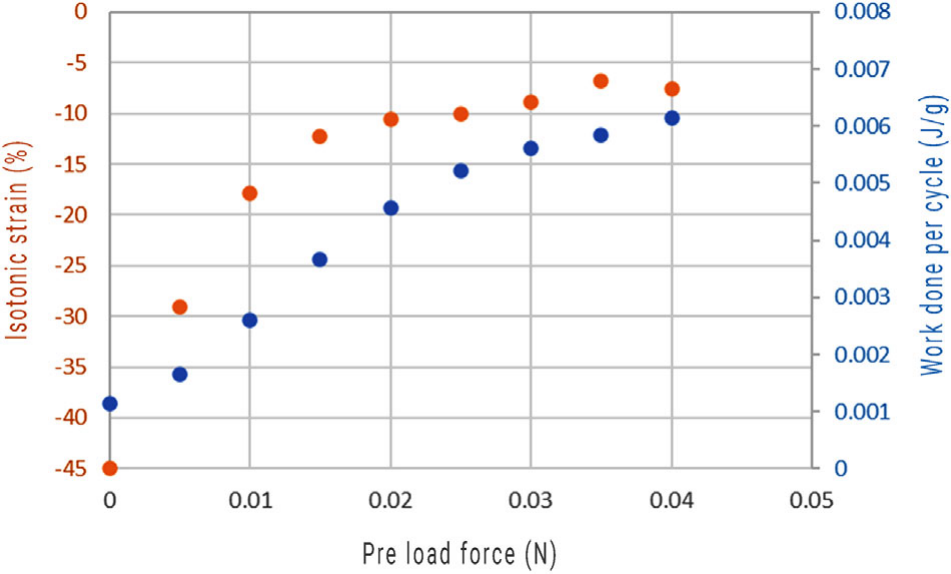
Isotonic strain and work output as a function of preload force for gel‐yarn composite actuator. Isotonic strain (%) and work done per cycle (J g^−^^1^) plotted against preload force (N) for a representative composite sample prepared with 1 M FeCl_3_. The isotonic strain was calculated from the difference between the actuated (swollen, 25°C) and unactuated (deswollen, 50°C) lengths at each preload condition. The work done per cycle was determined from the product of preload force and isotonic displacement. Data are derived from processed load‐extension curves.

### 3.7 Comparative Actuation Performance of Gel‐Yarn Composites

The actuation performance of gel‐yarn composite samples prepared with varying FeCl_3_ concentrations is summarized in Table 2. The maximum isotonic strain, work per cycle, and stiffness were determined for each sample. The 2 M FeCl_3_ samples exhibited the highest stiffness values, in the range of 0.05–0.09 N mm^−^^1^, but showed the lowest isotonic strains (3%–9%) and the lowest work per cycle (0.0003–0.00056 J g^−^^1^). In contrast, samples prepared with 0.1–1 M FeCl_3_ displayed isotonic strains in the range of 15%–39% and work per cycle values up to approximately 0.01 J g^−^^1^. The 1 M FeCl_3_ samples exhibited isotonic strains between 30% and 39%, while the 0.25 M samples showed values between 16% and 33%. The 0.1 M samples displayed isotonic strains ranging from 15% to 36%. Samples without FeCl_3_ (0 M) exhibited isotonic strains of 12%–20% and lower work per cycle values (0.0008–0.001 J g^−^^1^) compared to the intermediate concentration samples. The relationship between crosslinker concentration and work per cycle is shown in Figure 9. A variation in work output was observed across the tested concentrations, with lower values at higher FeCl_3_ concentration and higher values in the intermediate concentration range.

**TABLE 2:** Summary of actuation performance parameters for gel‐yarn composite actuators with varying FeCl_3_ crosslinker concentrations.

Sample	FeCl_3_ Conc. (M)	Max. Isotonic Strain (%)	Work per Cycle (J g^−^^1^)	Stiffness (N mm^−^^1^)
A	2	9	5.6 × 10^−^^4^	0.0467
B	2	3	3.0 × 10^−^^4^	0.0889
C	1	37	8.7 × 10^−^^3^	0.0072
D	1	30	8.8 × 10^−^^3^	0.0086
E	1	39	6.7 × 10^−^^3^	0.0112
F	0.25	33	5.3 × 10^−^^3^	0.0131
G	0.25	16	3.3 × 10^−^^3^	0.0112
H	0.1	15	1.4 × 10^−^^3^	0.0142
I	0.1	36	3.5 × 10^−^^3^	0.0089
J	0	20	1.0 × 10^−^^3^	0.0105
K	0	12	8.0 × 10^−^^4^	0.0264

**FIGURE 9:**
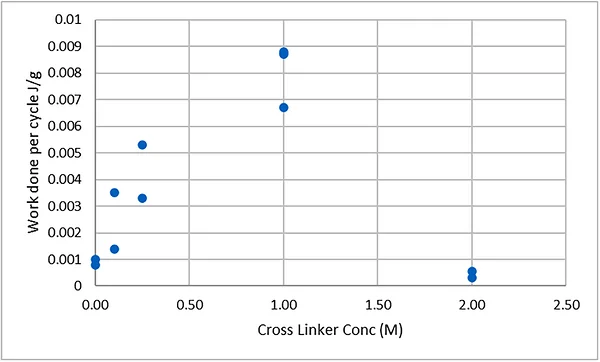
Effect of FeCl_3_ crosslinker concentration on the work per cycle (J g^−^^1^) of gel‐yarn composite actuators.

### 3.8 Scanning Electron Microscope (SEM) Analysis

Scanning electron microscopy (SEM) was employed to characterize the morphology of the gel‐yarn composites at multiple length scales. Representative images for samples prepared with 1 M and 0.1 M FeCl_3_, together with the yarn‐only control, are shown in Figure 10.

**FIGURE 10:**
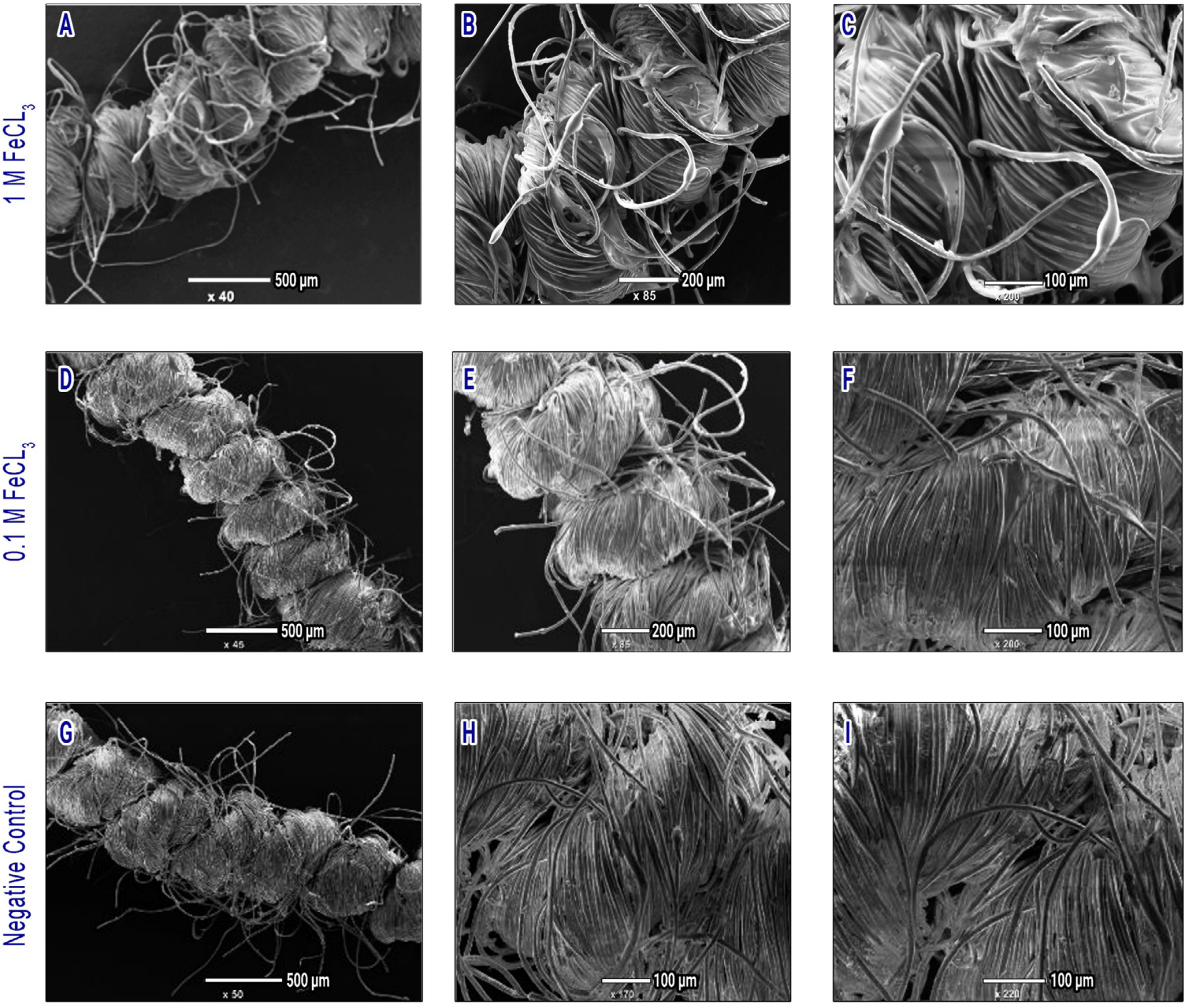
Scanning electron microscopy (SEM) images showing morphological comparison of gel‐yarn composites and control yarn at different magnifications. (A–C) Gel‐yarn composite prepared with 1 M FeCl_3_, showing (A) overall yarn structure, (B) fiber bundling with visible gel infiltration, and (C) higher magnification highlighting gel deposition on filament surfaces. (D–F) Composite prepared with 0.1 M FeCl_3_, showing reduced gel coverage and less uniform infiltration across the yarn filaments. (G–I) Control yarn (no FeCl_3_/polymer), displaying clean fiber morphology without gel coating. Scale bars: 500 µm (A, D, G), 200 µm (B, E), and 100 µm (C, F, H, I).

For the composite prepared with 1 M FeCl_3_ (Figure 10A–C), the low‐magnification image (Figure 10A) shows the twisted and coiled yarn architecture, with clearly defined fiber bundles forming the composite structure. At intermediate magnification (Figure 10B), the organization of individual filaments within the bundles becomes more apparent, with inter‐filament regions partially occupied by an additional phase. At higher magnification (Figure 10C), visible crust‐like regions of dried gel are observed within and between the filaments. These regions appear as irregular, continuous or semi‐continuous deposits bridging adjacent fibers and partially covering their surfaces. The presence of this material within the inter‐filament spaces indicates infiltration of the polymer solution into the yarn structure prior to gelation.

In the 0.1 M FeCl_3_ samples (Figure 10D–F), the overall yarn morphology and twisted fiber arrangement remain comparable to those observed in the 1 M samples. However, differences are evident in the extent and distribution of the gel phase. At similar magnifications, the inter‐filament regions appear less filled, and the visible crusts of dried gel are less pronounced and more discontinuous. The fiber surfaces remain more exposed, with fewer regions showing continuous coating or bridging between adjacent filaments.

The control yarn samples (Figure 10G–I), prepared without polymer solution or iron chloride, display the intrinsic morphology of the twisted yarn structure. At low magnification (Figure 10G), the coiled architecture of the fiber bundles is clearly observed. At higher magnifications (Figure 10H,I), individual filaments are well defined and largely free of surface deposits. No additional phase is observed within the inter‐filament spaces, and no crust‐like features are present.

Across all samples, the comparison of images at increasing magnification provides a consistent view of the fiber architecture and the presence or absence of an additional phase within the yarn structure. The composite samples exhibit visible gel deposition within the filament network, whereas the control samples show clean fiber surfaces without evidence of infiltration or coating.

### 3.9 Differential Scanning Calorimetry

The thermal behavior of equilibrated composite samples prepared with 1 M, 0.1 M, and 0 M FeCl_3_ was analyzed using differential scanning calorimetry (DSC). The samples were heated gradually to 50°C to identify the lower critical solution temperature (LCST), and the resulting thermograms are presented in Figure 11. The 0 M sample corresponds to a polymer‐only system without iron chloride.

**FIGURE 11:**
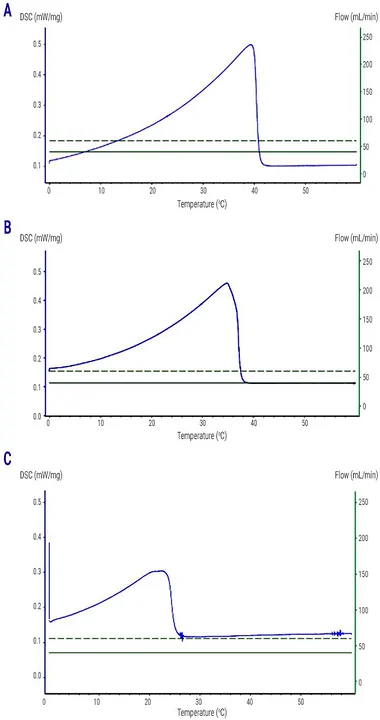
Differential scanning calorimetry (DSC) thermograms of hydrogel composites with varying FeCl_3_ concentrations. (A) 1 M FeCl_3_, (B) 0.1 M FeCl_3_, and (C) 0 M (polymer‐only) samples. All thermograms exhibit a thermal transition characteristic of LCST behavior. The transition temperature decreases with decreasing FeCl_3_ concentration, occurring at approximately 40°C, 37°C, and 25°C for 1 M, 0.1 M, and 0 M samples, respectively.

In all cases, a distinct thermal transition was observed, consistent with LCST‐type behavior. The transition temperature was found to decrease with decreasing iron chloride concentration, with transition points observed at approximately 40°C for 1 M, 37°C for 0.1 M, and 25°C for 0 M samples.

## 4 Discussion

The use of poly(MAA‐co‐OEGMA) in the gel‐yarn composite system enabled reversible and repeatable thermoresponsive behavior through swelling and deswelling transitions, both at the gel level and when integrated within fibrous architectures. While earlier thermoresponsive systems based on PNIPAM and Hydromed exhibited similar volumetric transitions, their translation into composite actuators often resulted in limited reproducibility and structural instability. However, the present system shows consistent actuation behavior, suggesting that effective coupling is achieved between the hydrogel phase and the fiber scaffold. In addition, the results indicate that introducing a hydrogel phase into a TCP structure can reduce the operating temperature required for actuation, addressing a key limitation of conventional systems that typically require temperatures exceeding 80°C [4, 5].

Beyond its swelling capacity, the poly(MAA‐co‐OEGMA) hydrogel contributes to structural stability through its intrinsic adhesive properties, which assist in maintaining the integrity of the twisted and coiled yarn configuration without the need for additional fixation materials. The incorporation of the hydrogel prior to twist insertion facilitates infiltration into the inter‐filament spaces, promoting physical integration of the polymer network within the fiber bundle and enabling mechanical coupling between the two phases. This integration provides a plausible pathway for transferring swelling‐induced stresses into macroscopic deformation; however, interfacial adhesion strength and stress‐transfer efficiency were not independently quantified in the present study. The mechanical robustness of the hydrogel can be attributed to its dual crosslinking characteristics, where ionic crosslinking through FeCl_3_ provides structural integrity, while reversible interactions such as hydrogen bonding between MAA and OEGMA segments enable energy dissipation under deformation [21, 24]. Similar dual‐network or sacrificial bond mechanisms have been widely reported to enhance toughness and fatigue resistance in hydrogel actuators [25, 26].

The mechanical response shown in Figure 3 reveals that ionic crosslinking produced a non‐linear trade‐off between stiffness, load‐bearing capacity, and reversible deformation. Increasing the FeCl_3_ concentration to 2 M substantially increased composite stiffness and maximum load; however, these samples generated only 3%–9% isotonic strain and 0.0003–0.00056 J g^−^^1^ of work per cycle. This behavior indicates that excessive ionic crosslinking restricted the network deformation required for swelling‐driven actuation. In comparison, the 1 M composites achieved 30%–39% isotonic strain and 0.0067–0.0088 J g^−^^1^ of work per cycle, while maintaining sufficient mechanical integrity and generating forces of approximately 40 mN. Lower crosslinker concentrations retained appreciable deformation but produced lower and more variable work outputs, whereas the absence of FeCl_3_ reduced the consistency of repeated actuation, most likely because the polymer network lacked sufficient ionic stabilization. The 1 M formulation therefore provided the most favorable balance between stiffness, reversible deformation, and mechanical work. Although loading‐unloading hysteresis was evident in all conditions, the loop areas were not quantitatively integrated; therefore, concentration‐dependent differences in dissipated energy cannot be conclusively compared from the present data.

The actuation behavior of the composite system is governed by the thermally induced volumetric transition of the hydrogel. At lower temperature (25°C), swelling of the hydrogel induces radial expansion within the filament bundle, generating internal stresses that are relieved through torsional untwisting of the yarn, resulting in axial contraction of the coiled yarn. Upon heating to 50°C, deswelling reduces the internal pressure, enabling re‐twisting of the fibers and recovery of the original length of the coil. This mechanism reflects a geometric amplification effect, where relatively small volumetric changes at the material level are translated into significant axial deformation through the twisted and coiled architecture. Such behavior is consistent with previously reported twisted and coiled polymer actuators, where torsional strain storage and release underpin actuation performance [4, 5, 23]. In the present study, however, swelling pressure, yarn torsional strain, and the corresponding geometric amplification factor were not independently measured; therefore, the proposed coupling mechanism should presently be interpreted as a mechanistic framework rather than a fully predictive quantitative model. The reversible actuation observed across the tested cycles indicates retention of functional mechanical coupling under the short‐term experimental conditions. However, dedicated interfacial characterization and extended fatigue testing are required to establish the long‐term stability of the fiber–gel interface. Ionic crosslinking is likely to contribute to maintaining network integrity during these short‐term tests.

The actuation performance is strongly influenced by the crosslinking density of the hydrogel network. At high FeCl_3_ concentrations (2 M), the increased crosslink density restricts polymer chain mobility and limits the extent of swelling, resulting in reduced isotonic strain and low work output despite increased stiffness. In contrast, at low or zero crosslinker concentration (0–0.1 M), insufficient network cohesion leads to reduced mechanical stability and inefficient stress transfer, as evidenced by variability in cyclic response. Optimal performance is achieved at intermediate crosslinking concentrations (0.25–1 M), where a balance between structural integrity and network flexibility allows for substantial swelling‐deswelling transitions while maintaining mechanical robustness. The maximum isotonic strain of 39% and corresponding work per cycle (0.0067–0.0088 J g^−^^1^) observed for the 1 M FeCl_3_ formulation are consistent with the general physical framework of hydrogel actuation, in which performance reflects the interplay between osmotic swelling pressure and elastic restoring forces [27, 28]. These observations are in line with previous studies showing that moderate crosslinking densities are required to achieve both sufficient mechanical stability and effective actuation in hydrogel systems [29, 30]. From an energy perspective, the achieved work density is comparable to other hydrogel‐based actuators but remains lower than high‐performance TCP or electroactive systems, highlighting the need for improved energy conversion efficiency in fiber‐gel composite designs [28, 31].

The experimental results demonstrate a clear trade‐off between composite stiffness and actuation deformation. Samples prepared with 2 M FeCl_3_ exhibited the highest stiffness (0.05–0.09 N mm^−^^1^), but produced only 3%–9% isotonic strain and 0.0003–0.00056 J g^−^^1^ of work per cycle. By comparison, the 1 M FeCl_3_ composites achieved 30%–39% isotonic strain and approximately 0.0067–0.0087 J g^−^^1^ of work per cycle, indicating a more favorable balance between network rigidity and swelling‐driven deformation. These findings suggest that excessive ionic crosslinking improves stiffness and dimensional stability but restricts the volumetric transition required for actuation, whereas insufficient crosslinking compromises structural integrity and stress transfer. Although force generation was derived from the load‐extension curves, response speed was not independently quantified across the crosslinking conditions. Further time‐resolved testing is therefore required to determine how network density influences actuation kinetics.

To position the present gel‐yarn composite relative to recently reported actuator systems, Table 3 compares its performance with representative hydrogel and TCP artificial muscles. The composite achieved an isotonic strain of up to 39% between 25°C and 50°C, which is comparable to the contraction reported for recent linear low‐density polyethylene TCP actuators operating at temperatures of up to 60°C. However, these fully synthetic TCP actuators generated substantially greater stress and specific power, reflecting their higher load‐bearing capacity [32].

**TABLE 3:** Comparison of the present gel‐yarn composite with representative recent hydrogel and TCP actuators.

Actuator system	Stimulus and operating conditions	Actuation strain or deformation	Force or stress output	Work or power output	Response or cyclic performance	Ref.
Poly(MAA‐co‐OEGMA)/polyester gel‐yarn TCP composite ‐(present study)	Hydrothermal actuation between 25°C and 50°C	Up to 39% isotonic tensile strain	Isometric force determined from force‐displacement curves	Work density on the order of 10^−^^3^ J g^−^^1^	Experimental protocol used 40 min at 25°C and 20 min at 50°C	This work
LLDPE TCP artificial muscle	Thermally actuated; characterized between approximately 20°C and 60°C	>40% contraction	0.3 ± 0.2 MPa at rest length; tensile stress >8.8 MPa without damage	Specific power >220 W kg^−^^1^	Peak strain rate 8.3% ± 0.9% s^−^^1^	[32]
Temperature‐triggered inflatable hydrogel muscle	Hydrogel‐mediated pneumatic actuation; heated from 20°C to 70°C	Rapid radial expansion; final actuator volume reached 50 times its initial volume in configuration	Blocking force up to 0.45 N	Work capacity 27.2 J kg^−^^1^	Actuation completed within approximately 10 s; peak radial expansion speed 200% s^−^^1^	[33]
Sensing‐actuating asymmetric hydrogel muscle	Electrothermal p(NIPAm‐HEMA) hydrogel; 20°C–45°C	58% volume shrinkage	Not reported as a blocking‐force value	Not reported	Shrinkage within 8 s; recovery within approximately 60 s at room temperature; sensing response linear up to 50% strain	[34]
Nylon and PVDF TCP actuators under high‐cycle testing	Hydrothermal cycling between 15°C and 75°C	Stroke remained generally stable during cycling; absolute length showed configuration‐dependent creep or reverse creep	Not reported as the principal comparison metric	Not reported	10,000 cycles over 56 h, with 20 s per cycle	[35]

Recent hydrogel actuators have achieved markedly faster responses through architectural strategies that reduce diffusion limitations or introduce mechanical instability. A temperature‐triggered inflatable hydrogel muscle generated a blocking force of 0.45 N and a work capacity of 27.2 J kg^−^^1^, with rapid expansion at 70°C [33]. Similarly, an open‐pore p(NIPAm‐HEMA) hydrogel achieved 58% volume shrinkage within 8 s at 45°C, while also integrating strain sensing and programmable heating [34]. High‐cycle evaluation of conventional TCP actuators has further demonstrated stable stroke over 10,000 thermal cycles, although creep or reverse creep altered the absolute actuator length in some configurations [35].

These comparisons indicate that the principal advantage of the present composite is its combination of large reversible tensile strain and relatively low operating temperature. Nevertheless, its potentially diffusion‐limited response associated with hydrogel swelling, comparatively low force and work output, and uncharacterized long‐term cyclic durability remain important limitations. Further optimization of hydrogel porosity, fiber–gel coupling, gel‐to‐yarn ratio, twist density, coil pitch, yarn diameter, actuator dimensions, and thermal transport will therefore be required to improve response speed and load‐bearing performance. Because actuator geometry, deformation mode, loading conditions, and normalization methods differ among studies, the values in Table 3 should be interpreted as performance benchmarks rather than direct equivalents.

The relationship between stiffness and actuation further highlights the trade‐off inherent in hydrogel‐based systems. While higher crosslinking enhances stiffness and load‐bearing capability, it simultaneously reduces the degree of volumetric expansion that drives actuation. Conversely, lower stiffness enables greater deformation but limits the force output. This trade‐off explains the observed variation in work per cycle across different crosslinking conditions and is consistent with previous reports on hydrogel actuators, where osmotic pressure‐driven actuation is inherently limited by the elastic resistance of the network [22, 26]. Although the present system demonstrates robust and reversible actuation, the generated forces (∼40 mN) remain lower than those reported for some fiber‐reinforced or purely synthetic TCP systems, which can reach significantly higher force outputs due to their higher modulus and more efficient stress transfer mechanisms [4, 5].

The fabrication strategy plays a critical role in determining the overall performance of the composite actuator. The ribbon configuration, in which the hydrogel is introduced prior to twisting, enables uniform distribution and effective infiltration within the yarn structure. This appears to improve mechanical coupling between the hydrogel and fiber network, providing a pathway for stress transfer during swelling and deswelling. In contrast, post‐coating methods tend to produce surface‐localized gel layers with limited penetration into the filament bundle, reducing actuation efficiency. The importance of fiber‐matrix integration in composite actuators has been widely recognized, with effective interfacial bonding being essential for translating material‐level responses into macroscopic actuation [18]. Although the present microscopy demonstrates physical integration of the gel within the fiber bundle, quantitative adhesion or interfacial shear measurements will be required to determine stress‐transfer efficiency in this system.

Thermal analysis further reveals that the lower critical solution temperature (LCST) of the composite system is influenced by crosslinker concentration, with increasing FeCl_3_ content leading to higher LCST values. This behavior reflects changes in polymer‐water interactions and network structure induced by ionic coordination, providing an additional parameter for tuning actuation behavior. The ability to modulate LCST through compositional control is a key advantage of poly(MAA‐co‐OEGMA)‐based systems compared to conventional PNIPAM hydrogels, which exhibit a relatively fixed transition temperature [19]. Morphological analysis via SEM confirms the presence of hydrogel within the filament bundles, with visible gel deposits bridging adjacent fibers in crosslinked samples. The reduced gel coverage observed at lower crosslinking concentrations is consistent with the reduced mechanical stability and actuation performance of these samples.

When compared to existing actuator systems, the present gel‐yarn composite shows a notable reduction in operating temperature while maintaining significant actuation strain. Conventional TCP actuators typically require temperatures exceeding 80°C to achieve large deformations [4, 5], whereas the present system operates effectively within a 25°C–50°C range. While the generated forces remain lower than those reported for fully synthetic fiber‐based systems, the reduction in operating temperature represents a meaningful trade‐off for applications where thermal constraints are critical.

Compared to PNIPAM‐based hydrogel actuators, which often suffer from low mechanical strength and limited force output [16, 17], the incorporation of a fibrous scaffold enhances structural stability and enables more efficient conversion of swelling‐induced stresses into mechanical work. However, the overall force output remains lower than that of fully synthetic fiber‐based actuators, indicating that further optimization of fiber‐gel interactions and network architecture is required. Similar challenges have been reported in hydrogel‐based actuation systems, where diffusion‐limited swelling and low modulus constrain performance [22, 26].

Despite these promising results, several limitations should be considered. The reliance on water‐mediated swelling introduces constraints related to environmental conditions and response kinetics, with equilibrium swelling times suggesting diffusion‐limited behavior that may restrict rapid actuation. Additionally, the relatively low force output limits applicability in load‐bearing systems, and long‐term cyclic stability and fatigue behavior were not extensively evaluated. Further work is therefore needed to improve force generation, reduce response times, and assess long‐term durability under repeated actuation cycles. Addressing these challenges will require optimization of hydrogel composition, crosslinking strategies, and fiber‐matrix interactions, as well as exploration of alternative stimuli or hybrid actuation mechanisms.

## 5 Conclusions

This work demonstrates a thermoresponsive gel‐yarn composite actuator capable of generating significant and reversible deformation at reduced operating temperatures. By integrating poly(MAA‐co‐OEGMA) hydrogel within twisted and coiled polyester yarns, the system achieves isotonic strains of up to 39% under a modest thermal stimulus (ΔT = 25°C), with an operating range between 25°C and 50°C.

The incorporation of ionic crosslinking enabled effective tuning of mechanical and actuation properties, with 1 M FeCl_3_ identified as the optimal concentration for balancing structural integrity, deformation, and work output. These results show that fiber‐gel integration can be used to adjust the actuation behavior of TCP systems without relying on high temperatures.

While the force output remains lower than that of purely synthetic fiber‐based actuators, the combination of low‐temperature operation, large deformation, and reversible behavior makes this approach particularly relevant for applications where thermal constraints are important. Future work should focus on improving actuation efficiency, response time, and long‐term stability. These improvements in performance would be aided by a quantitative model linking gel swelling to torsional strain and axial contraction. Further work could include simultaneous measurement of yarn rotation and coil geometry during gel swelling to validate such quantitative models.

The ability to achieve large, reversible deformation at relatively low temperatures positions these gel‐yarn composite actuators as promising candidates for applications in soft robotics, particularly in temperature‐sensitive environments. Potential applications include wearable assistive devices, biomedical systems, and microfluidic actuation platforms, where low thermal input is desirable. The biocompatibility and tunability of poly(MAA‐co‐OEGMA) further support its integration into bio‐interfacing systems. Moreover, the fiber‐based architecture offers scalability and compatibility with textile manufacturing processes, enabling the development of smart fabrics and distributed actuation systems. These features highlight the broader potential of gel‐yarn composite actuators as a versatile platform for next‐generation soft robotic technologies.

## Author Contributions

**Javad Foroughi**: conceptualization, investigation, funding acquisition, Writing – original draft, Writing – review and editing, validation, project administration, resources, supervision, data curation. **Sinmisola Aloko**: conceptualization, investigation, writing – original draft. **Geoffrey Spinks**: funding acquisition, writing – review and editing, conceptualization, project administration, resources, supervision, data curation, formal analysis, software, validation, methodology.

## Conflicts of Interest

The authors declare no conflicts of interest

## Data Availability

The data that support the findings of this study are available on request from the corresponding author. The data are not publicly available due to privacy or ethical restrictions.
